# A Comparison of the Present with the Past

**Published:** 1867-02

**Authors:** M. S. Dean


### A COMPARISON OF THE PRESENT WITH THE PAST.

BY M. S. DEAN.

Read before the Illinois State Dental Association.

Mr. President and Gentlenen:—The first course served at dinner usually consists of some variety of fish or soup. Whether this simpler food is given to the stomach to enable it to master and digest the rich and luxurious viands which are to follow, or whether it is sent to that organ as the *avant courier* to herald the coming of roast beef, turkey and other fat and distinguished guests, I know not with certainty, but believe the latter to be its true office. A glance at the *mental* bill-of-fare prepared by the committee will show that I am expected to supply that plain dish for this occasion, and that it holds the same relation to the intellectual feast which is to follow, as does fish or soup to the more substantial meal. This is all very proper, and is, I think, as it should be; but not being a practical caterer, and not knowing which of the two I can prepare the best, nor which of the two you might like the best, I will offer you a small quantity of fish-chowder, as *that* will serve the purpose of either one, or both combined, as may best suit your tastes. I am not entirely unselfish, I must confess, in offering you this dish; for notwithstanding they (Down East) would have you believe that it requires great skill to make a chowder, I assure you that it is the easiest dish to prepare in the whole range of cookery—fish, pork, potatoes, onions, and any thing else that may be found lying around loose, constitute its chief ingredients. For this last reason—as it will allow me to throw in anything that comes within my reach, without regard to the strict rules of culinary art—I shall attempt to provide this omnigenous dish, and, though you may find ingredients therein which may *appear* foreign to it, I hope you may not find it entirely unpalatable.

The assembling to-day of many of the most distinguished Dentists of our State, shows conclusively that they are not entirely satisfied with their present professional acquirements, notwithstanding they have already accomplished results, which, but a few years ago, would have been considered impossible, or which, when achieved, they might have reasonably deemed the very perfection of Dental art. These wonderful improvements, which have been developed, one after another, with astonishing rapidity, so far from satiating or even abating their desire for still greater knowledge of their profession, only stimulate and encourage them to explore still farther into this new, interesting, and useful field of science.

Other professions, learned and honorable, date their birth far back in the remote ages of the past, and have grown with the growth of civilization, and matured and ripened slowly; while ours seems almost to have leaped forth at a single bound, as did Minerva from the cleft brain of Jupiter. Indeed, so sudden has been its growth that the fact can scarcely be realized, even by the members themselves. The only means of bringing vividly before the mind its wonderful and unprecedented progress, is by comparing the mechanical branch of the past with that of the present time. A block of ivory, wrought with much patient labor and considerable skill, 'tis true, was worn as the substitute for natural teeth, and embodied all the skill and ingenuity that the profession possessed, only a few years ago. In the other departof our specialty, the progress, though not so capable of demonstration, has been none the less rapid and startling. In fact, the operative, surgical and medicinal branches may be considered the file leaders in the onward and victorious march of our calling.

The main causes which conspired to develop thus rapidly the germ of our profession, are really the *indirect* and less obvious ones.

The emigrants to our country, from its discovery to the present day, have suffered more or less by the change of climate; the sudden development of our agricultural domain; the sweeping away of our vast forests, and the cultivation of our boundless prairies—thus exposing, by the axe and the plow, measureless tracts of rich vegetable soil to the blazing rays of the sun—these, and the stagnant waters of our swamps and marshes, have filled the air with seeds of febrile disease, and produced their effects upon the entire population, both native and foreign, deranging the functions of the body, and vitiating the secretions. Add to these sources of disease, the medicines commonly exhibited in these fevers, of which mercury, in some of its forms, has been the chief, but by no means the only offender; an improper diet; hot, strong and stimulating drinks; the fatigues, exposure and privations to which the settlers of a new country are subjected—these, without naming the many lesser disturbing influences which derange the animal economy and deprave the fluids—these have been the prominent *predisposing* or *indirect* agencies which created the necessity for our profession in the country which may be considered the *mother* of Dentistry—America. But fortunately, the *force* of this necessity brought the art into living, active existence at that most favorable period in the world's history, when all the arts and sciences were cultivated to an extent never before known, furnishing, by ever-multiplying new discoveries, abundant materials already prepared for the experiments of the Dentist, and known scientific principles for his guidance; so that the founders of our profession needed not at first to *create*, but might freely select whatever was best adapted to their purpose. But, notwithstanding these favorable influences which surrounded its origin, and fostered its growth, it seems almost miraculous that our profession, so young, should take the respectable place that it does—though by no means a high one—among the learned professions which have struggled on for centuries, encountering and removing the obstacles which lay in their path, comparatively unaided. Nor could ours have attained this position, even by the aid of these *outside* influences, had there not been men *in* the profession not only skilled in the arts, but who, for scientific knowlege and *genius* stood unrivaled by those of any other profession, and who, by encouraging and educating the less disciplined members, brought it to its present degree of usefulness and eminence. Shall we rest content with this reputable position which our profession at present occupies or shall we raise it to the place which it ought to hold, and which its importance forcibly demands? Its usefulness no longer remains a matter of doubt, and its permanence is beyond a peradventure, for its existence must be co-extensive with civilization itself. The field, too, of our professional labor is boundless—it cannot be limited to a particular locality, country or continent, nor can its benefit be confined to a single class, community or race—they must be bestowed upon the inhabitants of the whole civilized world! Ought we not, in consideration of these facts, to fit ourselves to occupy a prouder and more useful position by raising *ours* to an equal rank, at least, with the other learned professions? If so, it can be done only by erecting a higher standard of education. By this means we better serve the interests of our patients, secure greater profit to ourselves, and higher honor to our profession. Is not then our specialty worthy of this promotion?

Is it not as necessary to the comfort and happiness of mankind as is that of surgery or medicine? Is not the class for which we labor as wealthy, as cultivated and as refined? And does it not require as thorough and as extended a course of study to prepare ourselves *properly* for this, as it does for those professions which we have just named?

These queries are all answered emphatically in the affirmative. Such, then, being the responsible position which we hold in the community, it becomes imperatively necessary that we improve all the aids which the sciences can afford us, in order that we may be completely armed to battle successfully those diseases which have hitherto defied all our skill.

Our professional education, instead of being superficial, or derived solely from a few works devoted specially to our calling, should be broad and substantial in its nature, and *threefold* in its character. The *mind*, the *hand* and the *eye* should each be schooled in its respective department. In other words, our education should be *scientific*, *mechanical* and *artistic*. The sciences of Anatomy, Physiology, Pathology and Chemistry should be as familiar to the Dentist as to the Physician. Nor should the nature and properties of medicines remain vague and mysterious agencies, but certain and potent remedies in our hands; for Therapeutics has already became an important arm of our profession, and is worthy of our most careful attention. The mind thus stored with varied knowledge, enables us to treat the diseases of the teeth themselves understandingly, and to discover the relations which certain diseases manifest towards them, or which may arise from sympathy with other affected organs. It also enables us to determine or disclose to view the many occult influences which the diseased teeth maintain towards the remote parts of the body, as well as to those more adjacently situated. The whole system, with its intricate and delicate functions, must be well understood in order that one of its important organs may be comprehended and successfully treated. In short, the science of medicine (in a restricted sense, perhaps), should be made the ground-work upon which our specialty is reared. For it will be readily agreed to, that the Dentist who has first laid within himself the foundation of a general knowledge of matters which relate to his profession, will be found the better qualified to practice it according to the light of reason and science.

The *hand*, also, must be thoroughly trained in mechanics, and in the handling of instruments properly and dexterously. It must be able to construct whatever the mind conceives, accurately and expeditiously; for the Dentist is often called upon to perform manual operations which are extremely difficult in their nature, and which require that precision and delicacy of touch which can only be acquired by long experience and the skillful training of the hand. If the hand lack the manipulative skill to execute whatever is prompted by the educated mind, both are not only practically useless, but productive of harm rather than good. If, therefore, the education of the hand be neglected, the Dentist, whose mind is fully stored with scientific knowledge, and who is endowed with the rarest gifts of genius to conceive or invent, is worthless in the most important branches of his profession; for the *hand* and *brain* must work harmoniously together.

The *education of the eye* is likewise of manifest importance to the *artistic* Dentist. By this external visual sense, the intellect becomes acquainted with the objects that surround us, and, from it, our operations derive all the artistic beauty which they may possess. The Dental Art, particularly, cannot be confined within the strict limits of rules. The correct form, arrangement, color and expression of artificial teeth can be determined by the educated eye alone. That it may be rendered exceedingly acute and delicate by education, is a physiological fact well established, and which is, by our experience or observation, fully verified. By this means, it becomes highly skillful in *taking impressions*

of nature, and in *seeing* that they are faithfully imitated in the work of the hand. It also holds within itself a true model of whatever the inventive mind conceives, while it guides and assists the hand in copying it correctly.

Although the mechanical operations of our profession (so far as they relate to artificial dentures), derive all their natural and lifelike character from the educated or artistic eye, yet teeth may be inserted without this artistic aid which will answer the useful purpose of mastication, and which are beautiful in their mechanism, when considered as such, but when viewed *in the mouth*, they neither resemble the living organs, nor restore the natural expression to the features. And yet, such teeth possess the virtue of *honesty*, as they would not deceive the most unsuspecting countryman as to their true character. These artificial teeth can be obtained more cheeply, of course, than those upon which not only mechanical but a higher order of skill has been expended; for the artistic eye hinders the rapid progress of the hand, and makes it work cautiously, and copy faithfully the natural organs. Since the introduction of vulcanized rubber as a base, teeth *generally* have been inserted so cheeply that if other than mechanical skill has been employed, it has been more the work of love than of compensation. For this reason, the artificial branch of our profession, which, for fifteen years previous, had made such unparalleled progress, has, for the last five or six years, been checked in its onward career. This is, however, only *generally* true of the profession; for there are many honorable exceptions to the rule. Teeth are inserted to-day which rival in beauty and naturalness of expression those of last year, and which challenge the closest scrutiny to detect them from the living ones; showing conclusively that there *are* men of genius devoting their talents to this important branch, and forcing it on to the highest degree of excellence, notwithstanding the many barriers in the guise of "*cheap work*" which they have to overcome—accomplishing results which can be attained only by the aid of the *educated hand* and *the educated eye.*

Nor does the importance of the artistic eye rest solely with this branch of Dentistry. The operator on the living teeth is thereby assisted in restoring them to their natural shape and beauty, and in removing or hiding the scars of battle which they may have received in their encouters with Dentist and Decay.

But, Mr. President and Gentlemen, when the Dental student has acquired, from a long term of pupilage, and from our colleges of learning (as far as he is capable), this scientific, mechanical and artistic knowlege, and has commenced his professional duties, he has but just entered upon his course of study, and investigation. If, from a feeling of self-sufficiency, he should hide himself within a crust of egotism, neither imparting light to, nor receiving it from, the experience of his professional brethren; if he should not avail himself of the advantages which arise from a free interchange of ideas, such as our Dental associations afford, and which are found to be so profitable to all who mingle in them; if he lack the aid, the stimulus, and the propulsive power which these societies impart—then, notwithstanding his educational advantages, he will soon find himself outstripped in the career of practical usefulness by those who started with far less scientific knowledge, but who have availed themselves of these manifestly needful auxiliaries. But this is hardly a supposable case, for those who are the best grounded in Dental knowlege, and those who have became eminent for their skill and genius—those, in fact, who require the least, because they have acquired the most, are among the first to establish these societies, and the very last who would be deprived of that kind of knowledge which can be obtained better from them than from any other source —I mean that knowlege which is derived from *comparison.* As some one before has said, comparison may be fairly termed the pioneer of all certain knowledge. Whether this be true or not, in a general sense, it is eminently true in regard to our specialty. For the purpose of securing this knowledge, the Illinois State Dental Society was formed, and to this end. each individual member should communicate freely and unreservedly his mode of practice; his successes and (if he have any) his failures; for by comparing these with our own, our judgment will be vastly aided in arriving at correct conclusions, and in determining and directing our future onward course. With all the subsidiaries we can bring to our assistance, our labors to perfect our profession must progress more and more slowly as it *approaches* perfection. In this respect it is like a beautiful, though unfinished piece of statuary, which only approximates the ideal formed in the artist's mind. As the work of the sculptor approaches completion, he becomes more cautious and studied. His blows, which, at first, thickened the air with the flying fragments, and at each stroke developed more plainly the form imprisoned within, now fall almost like the silent snowflakes, and wear away the marble so imperceptibly that the assiduous labor of months produces less visible change than did the first chip that bounded from his chisel; and now to crown his labors with triumphant success, it requires prolonged mental exertion and determined, persevering genius. His breast must glow with the true promethean fire, while his inspired hand, like that of Pygmalion, gradually transmutes the cold and senseless marble into life and beauty! So with our profession—the nearer it *approaches* perfection, the slower will be its apparent progress; and however real and important to mankind, less discernable to our perceptions.

We need not, then, be discouraged if the Art of Dentistry should apparently make slow progress for the next few years, as compared with the speedy growth of its infancy. If the great body of Dentists, with conscientious and enlightened effort can make little perceptible advance, then we may feel sure that the Art is approaching its ultimate limit of perfection. Besides, we are not required, like the sculptor, to finish the work, or, at least, to finish it alone—each of us working by himself to perfect his own ideal. We are at liberty to call in all the aid which the ever-luxurious growth of Science and the Arts afford us. We may avail ourselves of every advance in the knowledge of the human frame and its workings which the busy multitude of physi ologists are every day making. We may employ every new mineral the miner may drag from its concealment in the bowels of the earth. We may make use of every new substance the chemist may bring to light by analysis, or create by combination. We may have the advantage of every remedial and anæsthetic agent that the insatiable curiosity of the traveler, the thirsty mind of the naturalist, the scientific investigation or homely experience of the physician may find or hit upon. All these are co-workers with us in the advancement of Science, and from them we may freely take. Let us then continue to work, conscientiously, diligently and intelligently. Let us spend without grudging the results of our investigations and experiments; make known with modest pride our successes, and set up for example our failures without embarassment. Let us seek and improve every opportunity like the present to compare ourselves and our work with our neighbors and theirs, not in a spirit of rivalry, or to exalt ourselves above the rest, or to gain a glittering notoriety that will put a few coppers in our pockets; but in the true spirit of lovers of Natural Science and the Liberal Arts, seeking either to teach or to learn, to give or to receive information, to lend or to borrow experience according as we may come in contact with those who are below us, or those who have gone beyond us in the generous race. So ever is true Science advanced, and so, and so only, be assured, will our Science and Art become what it should be—an unalloyed blessing to mankind, and its practice *a Liberal Profession.*

